# The Public Health: Does It Matter?

**Published:** 1922-06

**Authors:** 


### THE PUBLIC HEALTH: DOES IT MATTER?

IT seems a foolish question to put in these enlightened twentieth century days. Of course, one is told, Public Health matters. All the legislative measures and local activities in regard to health bear witness to its importance and to the growing public conscience on this question.

It would be a mistake to belittle the extent of the achievements in recent years in the domain of public health. But the public conscience, though growing, is lethargic and needs continually rousing, and in these days when it is urged that nothing else matters but Economy, it is impossible to emphasise too much the paramount importance of the health of the nation.

Clarion calls go forth from time to time. England is told to "Wake up." She does so—and goes to sleep again. The imagination is stirred on the question of the Great White Scourge of tuberculosis—until some new sensation comes along. With the horror of the Great War fresh upon us, and on the wave of deep gratitude to those who went through it all, we get busy with houses fit for heroes to live in—and the performance, compared with the promise, is something to make the angels weep.

The content of the words "Public Health" is vast. The powers of local authorities are vast. That there is overlapping both at the centre and at the periphery is common knowledge. One of the objects of the Ministry of Health was to get rid of this overlapping. It is true that its days are yet few, but the road before it is long—and well worth travelling—and at present the Minister is held up at the toll-gate.

Exactly how vast is the content of the words "Public Health" may be a matter of opinion. But it is already recognised as embracing questions of environment, of education, of the protection of the community against the carelessness of the individual, of the control of food supply. How many of the members of a community ever see or even hear of the report of the medical officer of health for their area? They understand vaguely that such matters as housing, treatment of infectious diseases, maternity and child welfare, food inspection, are dealt with in some fashion by the Council, but these occur to their consideration mainly as an unpleasant burden of rates, or an interference with their liberty.

It is when the line between preventive and curative measures is passed that the difference of opinion between what is legitimately the concern of the body corporate and what is not manifests itself. The National Health Insurance Acts are justified to the employers as a matter of the restoration of the working capacity of the employed person, rather than as a measure for the relief of his suffering. It would be interesting to have the views of our public health authorities as to the ultimate lines of development of public health, whether, *e.g.*, considered from the angle of restoration (or, in the case of the child, the promotion) of working capacity, it does not in effect include the whole domain of curative treatment and care by the general practitioner, by the nurse, by the dentist, by the specialist, by the hospital authorities. And in the foreground is the question of the care of the child. Where is the line to be drawn here between inspection and treatment? Is it a good thing that one body, the Public Health Committee, should interest itself in the infant, that the Education Authority should independently look after the health of the school child, and then that at the age of 16 he should start afresh under the medical care of the National Health Insurance authority?

These, of course, are far-reaching questions, in regard to which local health authorities may be expected to express themselves only in very guarded fashion. But in regard to powers already in their hands, such questions as the following arise:—

> What are the exceptional needs of your area?
>
> What special powers do you possess?
>
> What forms of health activity have you specially developed—or to what do you attach special importance?
>
> How are you getting along with housing, tuberculosis treatment, the treatment of venereal disease, with maternity and child welfare, with the control of the food supply, with stamping out infectious disease in any of its forms?
>
> Is the system of control by the Ministry of Health well conceived? Does it help or hinder you in your activities?

And so on.

We propose to set down in regard to some of these questions, in each area, information obtained at first hand by visits to the chairman of the Public Health Committee and the medical officer of health for some of the larger industrial districts. It is impossible when travelling through some parts of the country not to realise very vividly that the industrialism of the past, mainly of the Victorian era, has left a terrible heritage to be tackled by those now responsible for the public health. Properly conceived, there can surely be no more fruitful or honourable field of service for any man than the wise control and development of those activities, in the area in which he lives, upon which depend the health,—and, therefore, the happiness—of his neighbours. And in the appointment of the chairman of their Public Health Committee—free from any political considerations—and of their medical officer of health, it must be the concern of a public, themselves awakened to a fuller sense of the health needs of their own district, to see that they get men of vision, and of plan, who will wisely press forward every well-conceived form of health activity towards the day when they can say, "We put Health first, and Prosperity followed as a matter of course, and we are proud of the results of our administration."

